# Integrated malaria prevention in low- and middle-income countries: a systematic review

**DOI:** 10.1186/s12936-023-04500-x

**Published:** 2023-03-06

**Authors:** David Musoke, Edwinah Atusingwize, Carol Namata, Rawlance Ndejjo, Rhoda K. Wanyenze, Moses R. Kamya

**Affiliations:** 1 Department of Disease Control and Environmental Health, School of Public Health, Makerere University College of Health Sciences, Kampala, Uganda; 2 Department of Medicine, School of Medicine, Makerere University College of Health Sciences, Kampala, Uganda

**Keywords:** Integrated approach, Malaria prevention, Multiple methods, Low- and middle-income countries

## Abstract

**Background:**

As many countries aim to eliminate malaria, use of comprehensive approaches targeting the mosquito vector and environment are needed. Integrated malaria prevention advocates the use of several malaria prevention measures holistically at households and in the community. The aim of this systematic review was to collate and summarize the impact of integrated malaria prevention in low- and middle-income countries on malaria burden.

**Methods:**

Literature on integrated malaria prevention, defined as the use of two or more malaria prevention methods holistically, was searched from 1st January 2001 to 31st July 2021. The primary outcome variables were malaria incidence and prevalence, while the secondary outcome measures were human biting and entomological inoculation rates, and mosquito mortality.

**Results:**

A total of 10,931 studies were identified by the search strategy. After screening, 57 articles were included in the review. Studies included cluster randomized controlled trials, longitudinal studies, programme evaluations, experimental hut/houses, and field trials. Various interventions were used, mainly combinations of two or three malaria prevention methods including insecticide-treated nets (ITNs), indoor residual spraying (IRS), topical repellents, insecticide sprays, microbial larvicides, and house improvements including screening, insecticide-treated wall hangings, and screening of eaves. The most common methods used in integrated malaria prevention were ITNs and IRS, followed by ITNs and topical repellents. There was reduced incidence and prevalence of malaria when multiple malaria prevention methods were used compared to single methods. Mosquito human biting and entomological inoculation rates were significantly reduced, and mosquito mortality increased in use of multiple methods compared to single interventions. However, a few studies showed mixed results or no benefits of using multiple methods to prevent malaria.

**Conclusion:**

Use of multiple malaria prevention methods was effective in reducing malaria infection and mosquito density in comparison with single methods. Results from this systematic review can be used to inform future research, practice, policy and programming for malaria control in endemic countries.

## Background

In 2020, there was an estimated 241 million cases of malaria, and 627,000 deaths from the disease globally [1]. Africa is the continent with the highest burden of malaria, accounting for 95% of all malaria cases and deaths, with children under 5 years of age and pregnant women being most vulnerable [1]. Estimates of the true burden of malaria in low-and middle-income countries (LMICs) including in Africa have been difficult to obtain due to underreporting of malaria cases and deaths [2]. The current malaria burden could thus be much higher than the estimates suggest. In addition to its impact on morbidity and mortality, the occurrence of malaria results in vast social and economic consequences. The economic costs are directly related to seeking treatment or preventive measures, or indirectly related to low productivity due to absenteeism from school or work, and time lost caring for the sick [3, 4].

Globally, malaria prevention has mainly relied on mosquito vector control by using insecticide-treated nets (ITNs), particularly long-lasting insecticidal nets (LLINs), and indoor residual spraying (IRS) [1]. In many malaria endemic countries, a vast effort has been made by ministries of health and their partners to increase coverage and utilization of ITNs and IRS. These efforts include the provision of LLINs and IRS to most at risk populations, although universal coverage for these interventions has not been achieved [5]. However, global malaria burden has not decreased significantly in recent years despite the efforts of increasing ITN and IRS coverage [1, 6]. Countries that have recorded significant gains in malaria control, such as El Savador which was certified by the World Health Organization (WHO) in 2021 as malaria free have used comprehensive approaches [1]. Although global and national malaria vector control efforts have predominantly focused on ITNs and IRS, several other control strategies can be implemented at household level to reduce mosquito density. These control measures include improving housing quality to limit mosquito entry, larval source management, and minimizing the presence of mosquitoes in houses for example by using insecticide sprays [7].

The use of appropriate combinations of non-chemical and chemical methods of malaria vector control in the context of integrated vector management has been recommended by the WHO [8]. Indeed, a combination of malaria prevention strategies has been shown to have greater impact than single methods [9–11]. Integrated malaria prevention therefore is an innovative approach that advocates the use of several malaria prevention measures in a holistic manner at household and community levels [12, 13]. These measures include proven malaria control methods and other approaches known to reduce mosquito populations. The specific methods advocated in the integrated approach are: (1). sleeping under LLINs; (2). installing screening in windows, vents and open eaves to prevent mosquito entry into houses; (3). IRS; (4). improving housing structure to limit mosquito entry; (5). larval source management; 6). closing windows and doors at sunset to reduce mosquito entry into houses; (7). larviciding in large water pools of stagnant water; (8). topical and spatial mosquito repellents; (9). mosquito coils; (10). insecticide sprays [12]. Although these various methods to reduce mosquito populations and prevent malaria exist, it is not expected that all of them will be used in a household due to several reasons including high cost, labour intensity, and side effects related to those that are insecticide based.

There is increasing evidence on the benefits of using multiple malaria prevention methods in households and communities particularly ITNs and IRS in comparison with single methods [14]. However, several studies have employed other malaria prevention methods in the integrated approach such as improving housing quality [15] and larviciding in recent years [16] Despite this available literature, there is limited evidence synthesizing findings from studies that have used two or more malaria prevention methods holistically. In addition, it is important to establish which other malaria prevention measures beyond ITNs and IRS have been used, and their contribution to controlling the disease. The aim of this systematic review was therefore to collate and summarize the impact of integrated malaria prevention in low- and middle-income countries on malaria burden. The systematic review adds to the evidence on use of two or more malaria prevention methods beyond the commonly used ITNs and IRS in endemic countries.

## Methods

PubMed, CINAHL, Web of Science, Embase, Cochrane library, Scopus, and The Malaria in Pregnancy Consortium Library, thesis online, Google Scholar, OpenGrey, ProQuest, ClinicalTrials.Gov, PACTR registry, and World Health Organization International Clinical trials registry platform were searched for literature from 1st January 2001 to 31st July 2021. Integrated malaria prevention was defined as the use of two or more malaria prevention methods holistically. The primary outcome variables were malaria incidence and prevalence, while the secondary outcome measures were human biting and entomological inoculation rates, and mosquito mortality. This systematic review was conducted and reported in accordance with the Preferred Reporting Items for Systematic reviews and Meta-Analysis Protocols Guidelines (PRISMA-P) [17]. The review protocol was registered in the International Prospective Register of Systematic Reviews (PROSPERO), registration number CRD42021277364.

### Eligibility criteria

The inclusion criteria was developed using the Population, Exposure, Comparator, Outcomes, Study characteristics framework, and studies were included if they met the following criteria:

- *Study population:* Individuals of all ages.
- *Type of exposure:* The intervention was use of integrated malaria prevention defined as the use of two or more malaria prevention methods holistically at a household or in the community [12].
- *Comparator:* The comparator group were households, individuals or communities not using integrated malaria prevention (using a single or no malaria prevention method at all).
- *Outcomes:* The primary outcome was occurrence of malaria (incidence or prevalence). Secondary outcomes were related to presence of mosquitoes in houses (including human biting rates, entomological inoculation rates, mosquito deterrence (preventing mosquito entry into houses) and mosquito densities). Definition of outcomes were as provided/defined by authors of included articles.
- *Study designs:* All study designs were considered.
- *Context:* Only studies conducted in LMICs, as defined by the World Bank Gross National Income per capita, calculated using the World Bank Atlas method as of June 2021 [18] were considered. The review included only literature published in English for a period of 20 years (January 2001 to June 2021). This period was expected to enable access to sufficient and relevant literature on integrated malaria prevention in LMICs.
- *Exclusion*: Duplicate publications, systematic or narrative reviews, reviews, abstracts, letters to the editor, comments, case reports, conference presentations, and study protocols were excluded.

### Search strategy and information sources

Two reviewers (EA and CN) independently conducted searches in PubMed, CINAHL, Web of Science, Embase, Cochrane library, Scopus, and The Malaria in Pregnancy Consortium Library. Other sources included: thesis online, Google Scholar, OpenGrey, ProQuest, ClinicalTrials.Gov, PACTR registry, and WHO International Clinical trials registry platform. A comprehensive search strategy with key terms based on the study population, exposure, and outcomes of interest was developed in PubMed (Table 1) and adjusted to suit other databases. Controlled descriptors were used (such as MeSH terms and Boolean operators) to ensure a robust search strategy (Table 1).

**Table 1:** Search Strategy

Themes	Search items
Study population	All individuals
Exposure	(Integrated malaria approach) OR (integrated malaria control) OR (integrated malaria prevention) OR (multiple malaria prevention) OR (combination malaria prevention) OR (ITNs) OR (insecticide treated nets) OR (long lasting insecticidal nets) OR (long-lasting insecticidal nets) OR (LLINs) OR (mosquito nets) OR (indoor residual spraying) OR (IRS) OR (window screening) OR (draining stagnant water) OR (closing windows and doors) OR (mosquito larviciding) OR (mosquito repellent) OR (mosquito coils) OR (mosquito insecticide spray)
Outcome	(Malaria prevalence) OR (malaria incidence) OR (malaria case) OR (mosquito prevalence) OR (mosquito presence)
Filters	**Year of publication:** (January 2001 to June 2021) **Language:** English **Population:** Humans

The database searches were supplemented by screening the bibliographies of relevant original research articles and systematic reviews. The references of all publications identified in the primary search were inspected, and where necessary, authors of individual studies were contacted for more information or clarity.

### Study screening and data extraction

**Fig. 1:**
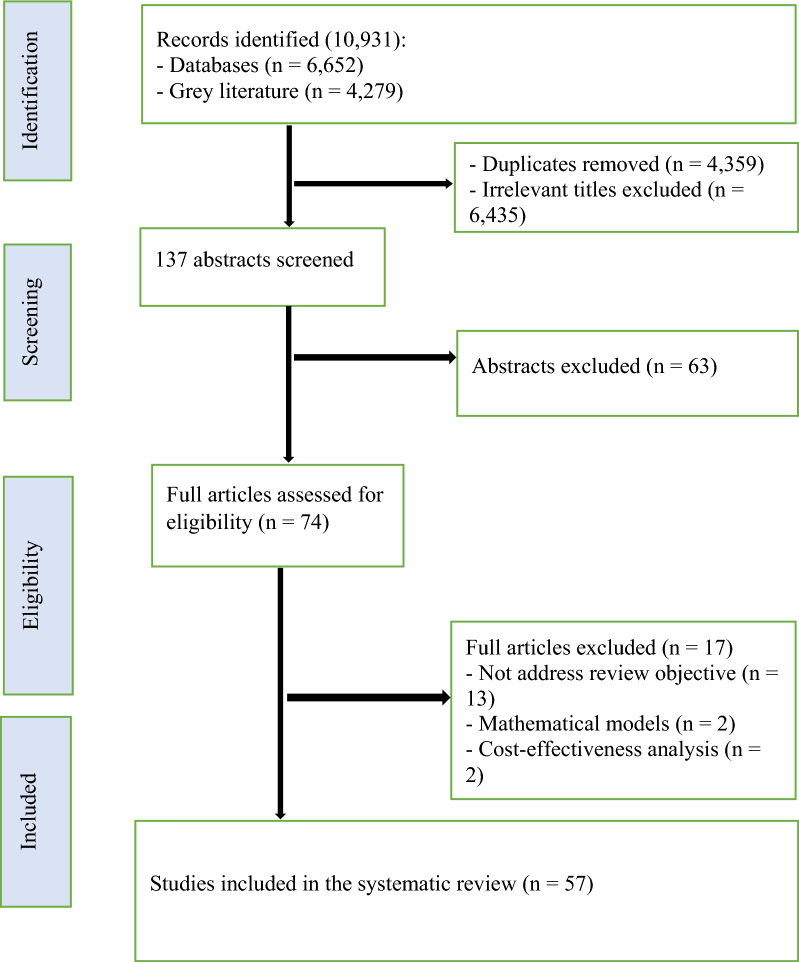
PRISMA flow diagram showing the selection process

The screening process was conducted at the title, abstract and full text levels by two reviewers (EA and CN) independently using defined criteria, and any discrepancies were resolved by consensus. Where necessary, the third reviewer (RN or DM) made the final decision, and all reasons for any exclusion of specific studies were documented. The study inclusion process was presented using the PRISMA flow chart (Fig. 1). Endnote reference management was used to store, organize, cite and manage all the included articles.

Data extraction was done independently by two members of the research team (EA and CN), extracting the relevant information from included full text articles onto an Excel spreadsheet, and later comparing and resolving any discrepancies. The specific data extracted included: country, design, participants/population, aim, intervention characteristics such as malaria prevention methods used, comparator group(s), duration, outcome measures, and main findings summary (Table 2). To avoid double counting, the results of studies presented in multiple papers for the same population were included once in the review.

**Table 2:** Characteristics of the included 57 studies

Author	Country	Study design	Study aim	Population	Intervention	Control	Duration of intervention	Outcome measures	Main findings summary
Sangoro et al. [21]	Tanzania	A cluster-randomized, placebo-controlled trial	To assess whether 15% DEET topical repellent used in combination with LLINs can prevent greater malaria transmission than placebo and LLINs	6 months and above	LLIN + DEET topical repellent	LLIN + placebo lotion	14 months	Rapid diagnostic test (RDT) confirmed malaria measured by passive case detection (PCD)	The placebo group comprised 1972.3 person-years with 68.29 (95% CI 37.05–99.53) malaria cases/1000 person-years The repellent group comprised 1952.8 person-years with 60.45 (95% CI 48.30–72.60) malaria cases/1,000 person-years, demonstrating a non-significant 11.44% reduction in malaria incidence rate in this group (Wilcoxon rank sum z = 0.529, p = 0.596)
Protopopoff et al. [22]	Tanzania	A cluster randomized controlled trial	To evaluate impact on malaria transmission comparing two rounds of IRS with bendiocarb plus universal ITN coverage, with ITNs alone	General population	IRS + ITN	ITN alone	21 months	Mosquito density monitored using CDC light traps during one night	Mean *An. gambiae s.l.* density in the ITN + IRS arm was reduced by 84% (95%CI 56–94%, p = 0.001) relative to the ITN arm. In the clusters categorised as high anopheline density at baseline EIR was lower in the ITN + IRS arm compared to the ITN arm (0.5 versus 5.4 per house per month, Incidence Rate Ratio: 0.10, 95% CI 0.01–0.66, p-value for interaction)
Okumu et al. [23]	Tanzania	Experimental study design (comparative field evaluation)	To evaluate combinations of LLIN and IRS, relative to either method alone, for malaria prevention in an area where the main vector is *Anopheles arabiensis*	Male volunteers aged between 18 and 35 years	LLIN + IRS or IRS + untreated nets	LLIN alone or untreated nets alone or unsprayed huts	13 months	Indoor mosquito bites, malaria vectors mortality, proportions caught exiting	All the net types, used with or without IRS, prevented > 99% of indoor mosquito bites. Adding Permanent 2.0 and Icon Life, but not Olyset nets into huts with any IRS increased mortality of malaria vectors relative to IRS alone. However, of all IRS treatments, only pirimiphos-methyl significantly increased vector mortality relative to LLINs alone, though this increase was modest. Overall, median mortality of *An. Arabiensis* caught in huts with any of the treatments did not exceed 29%. No treatment reduced entry of the vectors into huts, except for marginal reductions due to PermaNet 2.0 nets and DDT. More than 95% of all mosquitoes were caught in exit traps rather than inside huts
Maia et al. [24]	Tanzania	A household- cluster-randomized, placebo-controlled	To measure if diversion occurs from households that use repellents to those that do not use repellents	General population over 6 months	15% DEET + LLIN	Placebo	10 weeks	Mosquito densities	Repellent-users had consistently fewer mosquitoes in their dwellings. In villages where everybody had been given 15%-DEET, resting mosquito densities were fewer than half that of households in the no coverage scenario (Incidence Rate Ratio [IRR] = 0.39 (95% CI 0.25–0.60); p < 0.001). Placebo-users living in a village where 80% of the households used 15%-DEET were likely to have over 4-times more mosquitoes (IRR = 4.17; 95% CI 3.08–5.65; p < 0.001) resting in their dwellings in comparison to households in a village where nobody uses repellent
Killeen et al. (2017) [25]	Tanzania	Experimental study design	To assess window screens and eave baffles (WSEBs), which enable mosquitoes to enter but not exit houses, as an alternative to indoor residual spraying (IRS) for malaria vector control	General population	IRS using insecticides + LLIN + WSEBs	IRS using water + LLIN	Not clear	Mosquito vector mortality	Compared with IRS containing the same insecticide, WSEBs killed similar proportions of *Anopheles funestus* mosquitoes that were resistant to pyrethroids, carbamates and organochlorines and greater proportions of pyrethroid-resistant, early exiting *An. arabiensis* mosquitoes. WSEBs with pirimiphos-methyl killed greater proportions of both vectors than lambda-cyhalothrin or lambda-cyhalothrin plus pirimiphos-methyl and were equally efficacious when combined with binding agent. WSEBs required far less insecticide than IRS, and binding agents might enhance durability
Sternberg et al. [26]	Tanzania	Experimental study design	To evaluate “eave tubes”—a technology that combines house screening with a novel method of delivering insecticides for control of malaria mosquitoes	General population	LLIN + eave tubes (closed eaves and eave tubes treated with bendiocarb) or LLIN + open eaves	Untreated bednet (control group)	9 months	Larval densities, mosquito in-door host seeking densities	In the model village, introducing LLINs led to an approximate 60% reduction in larval densities and 85% reduction in indoor catches of host-seeking mosquitoes relative to pre-intervention values. Installing eave tubes and screening further reduced larval density (93% relative to pre intervention values) and virtually eliminated indoor host-seeking mosquitoes. When the eave tubes and screening were removed, larval and adult catches recovered to pre-eave tube levels
Protopopoff et al. [27]	Tanzania	A 4-group cluster randomised controlled trial using a two-by-two factorial design	To evaluate the effectiveness of Piperonyl butoxide (PBO) long-lasting insecticidal nets versus standard long-lasting insecticidal nets as single interventions and in combination with the indoor residual spraying of pirimiphos-methyl	Children aged 6 months to 14 years	Standard LLIN + IRS or PBO LLIN + IRS	Standard LLIN alone or PBO LLIN alone	34 months	Malaria infection prevalence	Malaria infection prevalence after 9 months was lower in the two groups that received PBO LLINs than in the two groups that received standard LLINs (odds ratio [OR] 0·37, 95% CI 0·21–0·65; p = 0·0011). At the same timepoint, malaria prevalence in the two groups that received IRS was lower than in groups that did not receive; OR 0·33, 95% CI 0·19–0·55; p < 0·0001) and there was evidence of an interaction between PBO LLIN and IRS (OR 2·43, 95% CI 1·19–4·97; p = 0·0158), indicating redundancy when combined. The PBO LLIN effect was sustained after 21 months with a lower malaria prevalence than the standard LLIN; OR 0·40, 95% CI 0·20–0·81; p = 0·0122)
West et al. [28]	Tanzania	A cluster randomised controlled trial	To investigate whether the combination provided added protection compared to ITNs alone	General population	ITN + IRS	ITN alone	48 months	*Plasmodium falciparum* parasite rate (PfPR) in children 0.5–14 years old, Anaemia in children 5 years old	In intention-to-treat analysis, mean PfPR was 13% in the ITN + IRS arm and 26% in the ITN only arm, odds ratio = 0.43 (95% CI 0.19–0.97, n = 13,146). The strongest effect was observed in the peak transmission season, 6 months after the first IRS. Subgroup analysis showed that ITN users were additionally protected if their houses were sprayed. Mean monthly entomological inoculation rate was non-significantly lower in the ITN + IRS arm than in the ITN only arm, rate ratio = 0.17 (95% CI 0.03–1.08)
De Castro et al. [29]	Tanzania	Case description of before and after	To describe and evaluate a control program that operated from 1988 to 1996 as a consequence of a bilateral agreement between the governments of Tanzania and Japan	General population	Chemical larviciding + Indoor residual house spraying (IRHS) + Space spraying of insecticides at ultra-low volume + ITNs + Environmental management	None, before and after data	96 months	Malaria prevalence, number of mosquito breeding sites	Reduced number of breeding sites. Malaria prevalence rates among school age children were reduced by approximately 50% over the eight-year period that the UMCP was operative (1988 – 1996)
Masalu et al. [30]	Tanzania	Experimental study design	To measure the additional benefits of combining transfluthrin-treated sisal decorations and LLINs with an aim of extending protection against early evening, indoor-biting malaria vectors when LLINs are ineffective	Male volunteers	Transfluthrin-treated sisal baskets + LLINs: (i) four transfluthrin-treated (2.5 ml) sisal baskets and one permethrin-treated LLIN; and (ii) four transfluthrin-treated sisal baskets (5 ml) and one permethrin-treated LLIN	Untreated sisal baskets + permethrin-treated LLIN	1 month	(i) Mosquito deterrence (reduction in the density of indoor mosquitoes) (ii) Indoor human mosquito biting rate (proportion of mosquitoes landing and attempting to bite volunteers) (iii) Insecticide-induced 24 h mortality	Sisal decorative baskets (0.28 m2) treated with 2.5 ml and 5.0 ml transfluthrin deterred 3/4 of Anopheles arabiensis mosquitoes from entering huts (relative rate, RR = 0.26, 95% CI 0.20–0.34, P < 0.001 and RR = 0.29, 95% CI 0.22–0.37, P < 0.001, respectively). Both treatments induced a tenfold increase in 24 h mortality of *An. arabiensis* mosquitoes (OR = 12.26, 95% CI 7.70–19.51, P < 0.001 and OR = 18.42, 95% CI 11.36–29.90, P < 0.001, respectively). Sisal decorative items treated with spatial repellents provide additional household and personal protection against indoor biting malaria and nuisance mosquitoes in the early evening, in absence of conventional indoor vector control tools
Menger et al. [31]	Kenya	Field experimental study design	To quantify the effects of eave screening in combination with a push–pull system based on the simultaneous use of a repellent (push) and attractant-baited traps (pull)	Male volunteers	Eave screening + outdoor, attractant-baited traps	No eave screening	33 consecutive nights	Mosquito house entry	Eave screening, whether used in combination with an attractant-baited trap or not, was highly effective in reducing house entry by malaria mosquitoes. The magnitude of the effect varied for different mosquito species and between two experiments, but the reduction in house entry was always considerable (61–99%). Using outdoor, attractant-baited traps alone did not have a significant impact on mosquito house entry but the high number of mosquitoes trapped outdoors shows that attractant-baited traps would enhance outdoor plus indoor protection against mosquito bites. As eave screening was effective by itself, addition of a repellent was of limited value, but could help in reducing outdoor malaria transmission in domestic areas
Mutero et al. [32]	Kenya and Ethiopia	A factorial, cluster-randomized, controlled trial	To assess the effect of supplementing LLINs with either larviciding with *Bacillus thuringiensis israelensis* (Bti) or community education and mobilization, or with both interventions in the context of IVM	School children	LLINs and Bti (arm 2); LLINs and community education and mobilization (arm 3); and, LLINs combined with Bti and community education and mobilization (arm 4)	LLINs only (arm 1)	36 months	Indoor mosquito density	There was no significant reduction in adult anopheline density at each of the three sites, which could be attributed to adding of the supplementary interventions to the usage of LLINs. Malaria prevalence was significantly reduced by 50% in Tolay when using LLINs coupled with application of Bti, and community education and mobilization. The two other sites did not reveal significant reduction of prevalence as a result of combining LLINs with any of the other supplementary interventions
Fillinger et al. [33]	Kenya	A controlled trial (pre-post, control group design was used)	To assess the contributions of both microbial larvicides and ITNs in terms of reducing malaria incidence in an integrated vector management programme in an area moderately endemic for malaria in the western Kenyan highlands	Children 6 months to 10 years old	Microbial larvicides + ITNs	ITNs alone	Not clear	Incidence of Plasmodium infections in children 6 months to 13 years of age	ITN use was associated with a 31% reduction in the risk of new malaria infections (OR: 0.69, 95% CI 0.48–0.99), while residence in an area with additional larviciding reduced the risk of new infections by 56% (OR: 0.44; 95% CI 0.23–0.82). Vector control with microbial larvicides enhanced the malaria control achieved with ITNs alone
Hamel et al. [10]	Kenya	A non-randomized prospective cohort study	To determine protective efficacy of IRS with ITNs compared with ITNs alone in preventing Plasmodium falciparum parasitemia	General population	IRS + ITNs	ITNs alone	Participants were followed for 1,197 person-years, 627 and 570 person-years in the ITN + IRS and ITN only groups, respectively	Incidence of P. falciparum parasitemia, Adjusted protective efficacy	Incidence of *P. falciparum* parasitemia in the ITN + IRS and ITN only groups was 18 and 44 infections per 100 persons-years at risk, respectively (unadjusted rate ratio = 0.41; 95% CI = 0.31–0.56). Adjusted protective efficacy of ITN + IRS compared with ITN only was 62% (95% CI = 0.50–0.72)
Gimnig et al. [34]	Kenya	Cross-sectional-Surveys	To evaluate the impact of an IRS program on malaria related outcomes in western Kenya, an area of intense perennial malaria transmission and moderate ITN coverage	General population	IRS + ITN	ITNs alone	24 months	Prevalence of malaria parasitemia, prevalence of clinical malaria	At baseline and after one round of IRS, there were no differences between the two districts in the prevalence of malaria parasitemia, clinical malaria or anemia. After two rounds of IRS, the prevalence of malaria parasitemia was 6.4% in the IRS district compared to 16.7% in the comparison district (OR = 0.36, 95% CI = 0.22–0.59, p < 0.001). The prevalence of clinical malaria was also lower in the IRS district (1.8% vs. 4.9%, OR = 0.37, 95% CI = 0.20–0.68, p = 0.001). Multivariate models incorporating both IRS and ITNs indicated that both had an impact on malaria parasitemia and clinical malaria but the independent effect of ITNs was reduced in the district that had received two rounds of IRS
Bousema et al. [35]	Kenya	A Cluster-Randomized Controlled Tria	To determine the impact of interventions targeted to serologically defined malaria hotspots on malaria transmission both inside hotspots and in surrounding communities	General population	Larviciding + LLINs + IRS + Focal mass drug administration	Malaria control following Kenyan national policy (IRS, routine case management at clinics, and bednet distribution at antenatal clinics)	7 months	Nested PCR (nPCR) parasite prevalence, Prevalence of clinical malaria, Mosquito density	Hotspot-targeted interventions did not result in a change in nPCR parasite prevalence outside hotspot boundaries (p ≥ 0.187). An average reduction in nPCR parasite prevalence of 10.2% (95% CI − 1.3 to 21.7%) inside hotspots 8 wk post-intervention was statistically significant after adjustment for covariates (p = 0.024), but not 16 wk post-intervention (p = 0.265). No statistically significant trend in the effect of the intervention on nPCR parasite prevalence in the evaluation zone in relation to distance from the hotspot boundary 8 wk (p = 0.27) or 16 wk post-intervention (p = 0.75). Thirty-six patients with clinical malaria confirmed could be located to intervention or control clusters, with no difference between the study arms. In intervention clusters, an average of 1.14 female anophelines inside hotspots and 0.47 in evaluation zones was caught; versus the control clusters with average of 0.90 female anophelines inside hotspots and 0.50 in evaluation zones, with no apparent difference between study arms
Okech et al. [36]	Kenya	Cross-sectional study	To investigate the potential factors that could have contributed to the decline of malaria cases in the hospital by analyzing the malaria control knowledge, attitudes and practices that the residents in Mwea applied in an integrated fashion	Children between 41/2 – 10 years	ITN + environmental management + mosquito repellent and smoke + insecticide canister sprays + window and door screens	None	Not clear	Malaria cases	Over the last 4 years prior to this study, the malaria cases in the community hospital reduced from about 40% in 2000 to less than 10% by 2004 and by the year 2007 malaria cases decreased to zero. The usage of a combination of malaria control tools in an integrated fashion by residents might have influenced the decreased malaria cases in the district hospital and in the school children
Bekele et al. [37]	Ethiopia	A cross-sectional study	To assess the effect of IRS and ITNs control strategies in Aneno Shisho kebele compared with Kamo Gerbi (supplied ITN only) and Jela Aluto (no IRS and ITNs), with regards to the prevalence of malaria and mosquito density	Under 5 years, and above 15 years	IRS + ITN	Single intervention	8 months	Malaria prevalence, Mosquito density	The difference in overall malaria prevalence and mosquito density between the three kebeles was significant (P < 0.05). The malaria prevalence rate in October/November 2006 in Jela Aluto, which was not covered by ITNs or IRS was significantly higher (10.4%) than Kamo Gerbi which was covered by ITN (5.4%) and in Aneno Shisho (1.7%), which was covered both by ITNs and IRS. In the under five years of age, malaria prevalence of October/November 2006 and April 2007 was 26.2% in Jela Aluto; 6.0% in Kamo Gerbi and 1.2% in Aneno Shisho. The difference in the under five years of age in prevalence among the three kebeles was statistically significant (P < 0.05). Malaria prevalence in the age group above 15 years was 9.5% in Jela Aluto; 10.2% in Kamo Gerbi and 3.7% in Aneno Shisho. The densities of *An. gambiae s.l.* were 11(73.3%) in Jela Aluto, 3(20%) in Kamo Gerbi and 1(6.6%) in Aneno Shisho
Asale et al. [11]	Ethiopia	A before and after cross-sectional study	To assess the impact of Integrated Vector Management (IVM) for malaria control in Botor- Tolay district, southwestern Ethiopia after three years (2016–2018) of IVM implementation	General population	Larva source management and environmental management + LLIN + IRS	None, before and after data	36 months	Malaria cases, Mosquito population	Significantly fewer adult mosquitoes were collected in 2018 (0.37/house/trap-night) as compared to 2015 (0.73/house/trap-night) (P < .001). Malaria cases significantly declined in 2018 (262) when compared to the record in 2015 (1162) (P < 0.001)
Deressa et al. [38]	Ethiopia	A community-based clustered-randomised trial	To determine the effect of combining community-based mosquito repellent with LLINs in the reduction of malaria	General population	Mosquito repellent + LLINs	Single intervention	4 months	Malaria infection	Compared with the control arm, the combined use of mosquito repellent and LLINs significantly reduced malaria infection of all types over time [OR = 0.66; 95% CI: 0.45–0.97]. Similarly, a substantial reduction in *P. falciparum* malaria infection during the follow-up surveys was observed in the intervention group (OR = 0.53, 95% CI 0.31–0.89). The protective efficacy of using mosquito repellent and LLINs against malaria infection of both *P. falciparum/P. vivax* and *P. falciparum* was 34–47%, respectively
Loha et al. [39]	Ethiopia	A 2 × 2 factorial, cluster-randomized, controlled trial	To evaluate whether the combined use of LLINs and IRS with propoxur provides additional protection against *Plasmodium falciparum* and/or *Plasmodium vivax* among all age groups compared to LLINs or IRS alone	General population	LLINs + IRS	LLINs alone OR IRS alone	29 months	Incidence of clinical malaria and anaemia prevalence	The overall malaria incidence was 16.5 per 1000 person-years of observation time, and similar in the four arms with 17.2 per 1000 person-years of observation time in the LLIN + IRS arm, 16.1 in LLIN, 17.0 in IRS, and 15.6 in the control arm. There was no significant difference in risk of anaemia among the trial arms
Musoke et al. [40]	Uganda	A qualitative cross-sectional survey	To assess the experiences of households using integrated malaria prevention as part of impact evaluation of the project 2 years after implementation	General population	LLIN + screening + removing mosquito breeding sites + closing of doors early	None, before and after data	12 months	Mosquito density, malaria prevalence	The major benefits reported from using integrated malaria prevention were reduction in mosquito populations in their houses and less occurrence of malaria especially among children
Katureebe et al. [41]	Uganda	Prospective observational study- before and after interventions-cohort studies, cross-sectional Community Surveys	To measure changes in key malaria indicators following universal LLIN distribution in three sites, with the addition of IRS at one of these sites	Children	LLIN + IRS	LLIN alone	28 months	Malaria test positivity rate (TPR), incidence of malaria, Human biting rate (HBR)	In Walukuba, over the 28-month post-intervention period, universal LLIN distribution was associated with no change in the incidence of malaria (0.39 episodes PPY pre-intervention versus 0.20 post-intervention; adjusted rate ratio [aRR] = 1.02, 95% CI 0.36–2.91, p = 0.97) and non-significant reductions in the TPR (26.5% pre-intervention versus 26.2% post-intervention; aRR = 0.70, 95% CI 0.46–1.06, p = 0.09) and HBR (1.07 mosquitoes per house-night pre-intervention versus 0.71 post-intervention; aRR = 0.41, 95% CI: 0.14–1.18, p = 0.10). In Kihihi, over the 21-mo post-intervention period, universal LLIN distribution was associated with a reduction in the incidence of malaria (1.77 pre-intervention versus 1.89 post-intervention; aRR = 0.65, 95% CI 0.43–0.98, p = 0.04) but no significant change in the TPR (49.3% pre-intervention versus 45.9% post-intervention; aRR = 0.83, 95% 0.58–1.18, p = 0.30) or HBR (4.06 pre-intervention versus 2.44 post-intervention; aRR = 0.71, 95% CI 0.30–1.64, p = 0.40). In Nagongera, over the 12-mo post-intervention period, universal LLIN distribution was associated with a reduction in the TPR (45.3% pre-intervention versus 36.5% post-intervention; aRR = 0.82, 95% CI 0.76–0.88, p < 0.001) but no significant change in the incidence of malaria (2.82 pre-intervention versus 3.28 post-intervention; aRR = 1.10, 95% 0.76–1.59, p = 0.60) or HBR (41.04 pre-intervention versus 20.15 post-intervention; aRR = 0.87, 95% CI 0.31–2.47, p = 0.80). The addition of three rounds of IRS at ~ 6-mo intervals in Nagongera was followed by clear decreases in all outcomes: incidence of malaria (3.25 pre-intervention versus 0.63 post-intervention; aRR = 0.13, 95% CI 0.07–0.27, p < 0.001), TPR (37.8% pre-intervention versus 15.0% post-intervention; aRR = 0.54, 95% CI 0.49–0.60, p < 0.001), and HBR (18.71 pre-intervention versus 3.23 post-intervention; aRR = 0.29, 95% CI 0.17–0.50, p < 0.001)
Rek et al. [42]	Uganda	Cohort study	To measure recent changes in house design in rural Uganda and evaluate their association with malaria in relation to a mass scale-up of control efforts	All children aged 6 months to 10 years	IRS + ITN + House improvements	ITN alone	74 months	Parasite prevalence, malaria incidence, HBR	IRS was associated with significant declines in human biting rate/HBR (33·5 vs 2·7 Anopheles per house per night after IRS, p < 0·0001), parasite prevalence (32·0% vs 14·0%, p < 0·0001), and malaria incidence (3·0 vs 0·5 episodes per person-year at risk, p < 0·0001). Compared with traditional houses, modern houses were associated with a 48% reduction in HBR before IRS (adjusted incidence rate ratio [aIRR] 0·52, 95% CI: 0·36–0·73, p = 0·0002), and a 73% reduction after IRS (aIRR 0·27, 0·17–0·42, p < 0·0001). Before IRS, there was no association between house type and parasite prevalence, but after IRS there was a 57% reduction in the odds of parasitaemia in modern houses compared with traditional houses (adjusted odds ratio 0·43, 95% CI 0·24–0·77, p = 0·004). House type was not associated with malaria incidence before or after IRS
Oguttu et al. [43]	Uganda	Observational retrospective analysis	To assess malaria incidence, test positivity rates and outpatient attendance due to malaria before and after vector control interventions	Children and adults	LLINs + IRS	LLINs alone	48 months	Malaria incidence, malaria test positivity rates	A rapid reduction in malaria incidence was observed in the district following the introduction of IRS in addition to LLINs. -Following universal LLINs coverage, the annual mean monthly malaria incidence fell from 95 cases in 2013 to 76 cases per 1000 in 2014 with no significant monthly reduction (OR = 0.99, 95% CI 0.96–1.01, P = 0.37). Among children < 5 years, the malaria incidence reduced from 130 to 100 cases per 1000 (OR = 0.98, 95% CI 0.97–1.00, P = 0.08) when LLINs were used alone in 2014, but declined to 45 per 1000 in 2015 when IRS was combined with LLINs (OR = 0.94, 95% CI 0.91–0.996, P < 0.0001). Among individuals aged ≥ 5 years, mean monthly malaria incidence reduced from 59 to 52 cases per 1000 (OR = 0.99, 95% CI 0.97–1.02, P = 0.8) when LLINs were used alone in 2014, but reduced significantly to 25 per 1000 in 2015 (OR = 0.91, 95% CI 0.88–0.94, P < 0.0001). Malaria test positivity rate reduced from 57% in 2013 to 30% (Chi = 15, P < 0.0001) in 2015. Slide positivity rate reduced from 45% in 2013 to 21% in 2015 (P = 0.004) while RDT positivity declined from 69 to 40%
Musiime et al. [44]	Uganda	A cross-sectional study before and after	To compare malaria transmission indoors and outdoors, before and after mass deployment of LLINs and IRS	General population	LLIN + IRS	LLIN	96 months	Human biting rate, annual entomological inoculation rate, proportion of mosquitoes collected outdoors	The interventions were associated with a decline in human biting rate from 19.6 to 2.3 female Anopheles mosquitoes per house per night (p < 0.001) and annual entomological inoculation rate from 129 to 0 infective bites per person per year (p < 0.001). The proportion of mosquitoes collected outdoors increased from 11.6 to 49.4% (p < 0.001). Before interventions, the predominant species was *Anopheles gambiae sensustricto* (s.s.), which comprised 76.7% of mosquitoes. Following the interventions, the predominant species was *Anopheles arabiensis*, which comprised 99.5% of mosquitoes, with almost complete elimination of *An. gambiae s.s*. (0.5%)
Galatas et al. [45]	Mozambique	A before and after cross-sectional study	To evaluate a multi-phased malaria elimination project to interrupt Plasmodium falciparum malaria transmission	General population	Mass Drug Administration + IRS + LLIN	None-before and after	Not clear	Malaria prevalence and incidence rates. Yearly parasite surveys and routine surveillance data were used to monitor the outcomes of the study at baseline and annually since the onset of the project	Parasite prevalence declined from 9.1% (95% CI: 7.0–11.8) to 2.6% (95% CI 2.0–3.4), representing a 71.3% (95% CI 71.1–71.4, p < 0.001) reduction after phase I, and to 1.4% (95% CI 0.9–2.2) after phase II. This represented an 84.7% (95% CI 81.4–87.4, p < 0.001) overall reduction in all-age prevalence Case incidence fell from 195 to 75 cases per 1000 during phase I (61.5% reduction) and to 67 per 1000 during phase II (65.6% overall reduction). Phase I interventions were associated with a significant immediate reduction in cases of 69.1% (95% CI 57.5–77.6, p < 0.001). Phase II interventions were not associated with a level or trend change. An estimated 76.7% of expected cases were averted throughout the project (38,369 cases averted of 50,005 expected)
Temu et al. [46]	Mozambique	Cross-sectional community-based surveys	To assess the impact of IRS and ITNs, the effects of keeping farm animals and of the construction material of roofs of houses and other potential risk factors associated with malaria infection in children	Children (ages 1–15 years)	LLIN + IRS	LLIN alone	36 months	Malaria prevalence, protective factors independently associated with malaria infection	Prevalence of malaria infection was 47.8% (95% CI 38.7–57.1%) in children 1–15 years of age, less than a quarter of children (23.1%, 95% CI 19.1–27.6%) were sleeping under ITN and almost two thirds were living in IRS treated houses (coverage 65.4%, 95% CI 51.5–77.0%). Protective factors independently associated with malaria infection included: sleeping in an IRS house without sleeping under ITN (OR = 0.6; 95% CI 0.4 – 0.9); additional protection due to sleeping under ITN in an IRS treated house (OR = 0.5; 95% CI 0.3–0.7) versus sleeping in an unsprayed house without a ITN was noted
Chaccour et al. [47]	Mozambique	A two-arm, cluster randomized, controlled study design	To provide evidence on the incremental epidemiological benefit of using third-generation IRS product in a highly endemic area with high ITN ownership	Children under 5 years were enrolled in the cohort	ITN + IRS	ITN alone	36 months	Malaria incidence rate, malaria prevalence	Children in the IRS arm experienced 4801 cases (incidence rate of 3532 per 10,000 children-month at risk) versus 5758 cases in the no-IRS arm (incidence rate of 4297 per 10,000 children-month at risk), resulting in a crude risk reduction of 18% and an incidence risk ratio of 0.82 (95% CI 0.79–0.86, p-value < 0.001). Facility and community passive surveillance showed a malaria incidence of 278 per 10,000 person-month in the IRS group (43,974 cases over 22 months) versus 358 (95% CI 355–360) per 10,000 person-month at risk in the no-IRS group (58,030 cases over 22 months), resulting in an incidence rate ratio of 0.65 (95% CI 0.60–0.71, p < 0.001). In the 2018 survey, prevalence in children under five in the IRS arm was significantly lower than in the no IRS arm (OR 0.54, 95% CI 0.31–0.92, p = 0.0241)."
Makoutode et al. [48]	Benin	A cross-sectional study	To see if adding the IRS to the LLINs (municipality of Kouandé) strategy is cost-effective, as compared to the LLINs-only strategy (municipality of Copargo)	General population	IRS + LLIN	LLIN alone	N/A	Cost-effectiveness ratio by malaria case, annual incidence of malaria	After LLINs + IRS intervention, (1) the annual incidence of malaria in health facilities decreased significantly at Kouandé-Centre. In the same period, it increased significantly at Copargo- Centre. (2) The average cost per malaria case prevented (CE) was respectively 85,572.4 FCFA at Copargo Centre, 38,932.6 FCFA at Kouandé Centre. The CE ratio at Kouandé-Centre was lower than the CE ratio at Copargo- Centre. The LLINs + IRS strategy was more cost effective in urban areas than the LLINs-only strategy. The opposite result was observed in rural areas
Corbel et al. [49]	Benin	A cluster randomised controlled trial	To Investigate whether the combination of LLINs with IRS or carbamate-treated plastic sheeting (CTPS) conferred enhanced protection against malaria and better management of pyrethroid-resistance in vectors than did LLINs alone	Pregnant women and children under 6 years	4 interventions 1- TLLIN (LLIN targeted coverage to pregnant women and children younger than 6 years), 2- ULLIN (LLIN universal coverage of all sleeping units), 3- TLLIN plus full coverage of carbamate IRS applied every 8 months (TLLIN + IRS), 4- ULLIN plus full coverage of CTPS lined up to the upper part of the household walls (ULLIN + CTPS)	TLLIN	18 months	Clinical incidence density of malaria in children younger than 6 years	The clinical incidence density of malaria was not reduced in the children from the ULLIN group (incidence density rate 0·95, 95% CI:0·67–1·36, p = 0·79), nor in those from the TLLIN + IRS group (1·32, 95% CI 0·90–1·93, p = 0·15) or from the ULLIN + CTPS group (1·05, 95% CI 0·75–1·48, p = 0·77) compared with the reference group (TLLIN). The same trend was observed with the prevalence and parasite density of asymptomatic infections (non- significant regression coefficients). Finally, fewer mosquitoes entered the houses containing both IRS + LLINs than houses with LLINs alone
Ngufor et al. [50]	Benin	Experimental study design	To compare the efficacy of Interceptor® G2 LN, a newly developed LN treated with a mixture of chlorfenapyr (a pyrrole) and alpha-cypermethrin (a pyrethroid), to a combined chlorfenapyr IRS and Interceptor^®^ LN (a standard alpha-cypermethrin LN) intervention	Human volunteer sleepers	ITNs + IRS	Untreated nets	4 months	Mosquito mortality, blood feeding inhibition,	Mortality in the control (untreated net) hut was 5%. Mortality with Interceptor^®^ LN (24%) was lower than with chlorfenapyr IRS alone (59%, P < 0.001). The combined Interceptor^®^ LN and chlorfenapyr IRS intervention and the mixture net (Interceptor^®^ G2 LN) provided significantly higher mortality rates (73 and 76%, respectively) and these did not differ significantly between both treatments (P = 0.15). Interceptor LN induced 46% blood-feeding inhibition compared to the control untreated net, while chlorfenapyr IRS alone provided none. Both mixture/combinations also induced substantial levels of blood-feeding inhibition (38% with combined interventions and 30% with Interceptor^®^ G2 LN). A similar trend of improved mortality of pyrethroid-resistant An. gambiae s.l. from Cove was noted with Interceptor® G2 LN (79%) versus Interceptor LN (42%, P < 0.001) in WHO tunnel tests
Aregawi et al. [51]	Ghana	Cross sectional study	To assess the impact of interventions on malaria cases, admissions and deaths using data from district hospitals	General populatoin	IRS, ITN, and artemisinin-based combination therapy	Non-IRS districts	10 years	TPR, malaria admissions, malaria deaths	Difference in IRS and non-IRS districts: In the regions where IRS was implemented during 2006–2015, the TPR in all ages decreased significantly by 89% (77–95%); malaria admissions and deaths decreased significantly by 68% (21–87%) and 88% (71–95%), respectively. The decrease in trends of malaria indicators in the non-IRS districts (34 within the same regions) was smaller. The TPR decreased only by 38% (16–54%); malaria admissions showed little change, 35% (− 15 to 63%), and malaria deaths decreased by 44% (16–62%). The decreases in proportion of malaria outpatients, inpatients and deaths of all-cause conditions were much higher in the IRS districts compared to the non-IRS district
Okyere [52]	Ghana	Cross-sectional study	To examine the interaction effects of household use of bed nets and insecticide products on self-reported malaria prevalence using panel data collected from two administrative districts and a doubly robust estimation technique	General population	ITNs + household insecticide products	ITNs alone	Not clear	Malaria prevalence	The use of bed nets was associated with lower malaria among household members. Household use of insecticide products singly shows no statistically significant negative relationship with malaria. Indicating some evidence that adopting the two measures jointly increases the efficacy of insecticide products—combining bed nets and household insecticide products reduce malaria for all individuals, females and children under five years
Afoakwah et al. [53]	Ghana	Cross-sectional study (data sourced from the current round of the Ghana Demography and Health Survey)	To investigate the association with use of large-scale malaria interventions such as: IRS, ITNs, and Behaviour Change Communication strategies, and the prevalence of malaria among children under-five in Ghana 2014 survey	Children under-five years	ITN and IRS	ITN alone OR IRS alone data	Not clear	Malaria prevalence	The dual use of both ITN and IRS, does not provide an added protection;—IRS offers much more protection than ITN use. The odds of malaria infection among children in IRS was significantly lower (OR = 0.312; 95% CI − 1.47—0.81; p = 0.00) compared to those not protected. This association was even high (OR = 0.372; 95% CI − 1.76—1.02; p = 0.00) among children in poor households protected by IRS compared to those with no IRS protection. ITN use did not have a significant association with malaria infection among children, except among children whose mothers had at least secondary education. For such children, the odds of malaria infection were significantly lower (OR = 0.545; 95% CI − 0.84—0.11; p = 0.011) compared to those who not protected
Prakash et al. [54]	India	Operational research study	To develop an effective malaria control strategy for the oil personnel working in these areas, a 1-year pilot study (April 2000 to May 2001) was carried out in the Jorajan camp of OIL	Oil employees	Deltamethrin-treated mosquito nets + Mosquito repellent cream + Weekly chemoprophylaxis with 300 mg chloroquine	None, before and after data	12 months	Mean landing rate of *Anopheles dirus*, incidence of malaria, malaria mortality	The mean landing rate of *Anopheles dirus*, the vector mosquito in the camp area, was 5.03 per person per night during monitoring. The incidence of malaria in the camp was reduced by > 90% as compared to previous years and the number of malaria cases came down from 6.7 per 1000 man-nights in 1998–99 to 0.06 in 2000–01. Mortality due to malaria was completely eliminated
Dutta et al. [55]	India	Household survey, surveillance, and Entomological and parasitological baseline and follow-up	To evaluate the preventive efficacy of ITNs and mosquito repellent in a malaria-endemic foothill area of Assam, India, with forest ecosystem, 2003 – 2006	General population	During the second year, intervention measures were implemented in the four sectors as follows: A- ITN + repellent B- ITN; C- repellent; D- Information, Education and Communication activities were carried out in sectors A, B and C	No intervention	36 months	Malaria protective efficacy, vector population	The most effective intervention was in sector A (ITN + repellent), followed by sectors B and C. Sectors A and B exhibited significantly higher (P < ;0.001) malaria protective efficacy during both the first and second years of intervention compared with sector D. The total vector population in the three intervention sectors decreased significantly compared with that of the non-intervention one
Singh et al. [56]	India	Cross-sectional surveys, pre- and post-intervention surveys	To assess the impact of intensified malaria control interventions in an ethnic minority community in Betul using existing tools	Children up to 10 years of age and an older age group > 10 years	2 rounds of IRS + larvivorous fish + intensive surveillance for early detection of *Plasmodium falciparum* with rapid diagnostic tests and prompt treatment with sulphadoxine pyrimethamine	None, before and after data	Not clear	Malaria cases, Mosquito population	Pre-intervention surveys revealed a very high fever rate in the community in all age groups with a slide positivity rate of > 50% with > 90% *P. falciparum*. The post-intervention phase showed a sharp steady decline in number of malaria cases (β 0.972; p < 0.0001, 95% CI 0.35–0.47). Monitoring of entomological results revealed a significant decline in both Anopheles species and *An. culicifacies* (p < 0.0001)
Nwaneri et al. [57]	Nigeria	A cross-sectional descriptive study	To document factors that influence regular use of ITNs in under-fives and impact of vector control methods on malaria outcome (severe malaria prevalence and mortality) in under-fives presenting in a tertiary health institution in Nigeria	Under 5 years	ITN + indoor insecticide spray + netting of doors/window + regular environmental sanitation (clearing bushes and drainages around the house)	None	14 months	Severe malaria prevalence and mortality in under-fives	Prevalence of severe malaria was 36.2% and mortality was 52 per 1000. Combination of regular use of insecticide treated nets, environmental sanitation, indoor insecticide spray and netting of household doors/windows significantly predicted low prevalence of severe malaria compared to each of the malaria vector control methods used singly by the caregivers (β = 1.66, OR = 5.0, p = 0.04)
Agomo et al. [58]	Nigeria	A cross-sectional study	To identify the factors associated with risk of malaria infection in pregnant women	Pregnant women	Insecticide spray + ITN	Single intervention	12 months	Malaria prevalence	Malaria preventive practices associated with a significant reduction (P < 0.05) in the malaria infection was the use of insecticide sprays (RR = 0.36, 95 CI 0.24—0.54), and the combined use of insecticide spray and insecticide-treated nets (ITN) (RR = 6.53, 95% CI 0.92–46.33). Sleeping under ITN alone (RR = 1.07, 95% CI 0.55–2.09) was not associated with significant reduction in malaria infection. Young maternal age (< 20 years) (RR = 2.86, 95% CI 1.48–5.50), but not primigravidity (RR = 1.36, 95% CI 0.90–2.05), was associated with an increased risk of malaria infection during pregnancy. After a multivariate logistic regression, young maternal age (OR = 2.61, 95% CI 1.13–6.03) and the use of insecticide spray (OR = 0.38, 95% CI 0.24–0.63) were associated with an increase and a reduction in malaria infection, respectively
Kawada et al. [59]	Malawi	A before and after cross-sectional study	To examine the effect of the combined use of metofluthrin-impregnated spatial repellent devices (MSRDs) and LLINs (Olyset® Plus) on malaria prevalence and vector mosquitoes were examined in malaria endemic villages in south-eastern Malawi	Children	MSRDs + LLINs (Olyset Plus)	LLINs alone (Olyset Plus)	24 months	Infection rate in children, Number of vector mosquitoes	The intervention reduced the infection rate in children as well as the No. pyrethroid resistant vector mosquitoes. In the preliminary field study, significant reduction in the number of mosquitoes was observed in the houses treated with MSRDs in combination with Olyset^®^ Plus compared to that of the control houses. The reduction in the number of mosquitoes was highest in the houses treated with Olyset^®^ Plus + MSRDs. Significant reduction in the number of mosquitoes compared to the control houses was observed at 3 months after the abovementioned intervention regimes were implemented, although the mosquito numbers slightly resurged after 3 months of intervention
McCann et al. [60]	Malawi	A two-by-two factorial, cluster-randomized controlled trial	To assess the effects of community-based house improvement and larval source management (LSM) as supplementary interventions to the Malawi National Malaria Control Programme interventions in the context of an intensive community engagement	General population	(i) Malawi National Malaria Control Programme (IRS + ITNs + intermittent preventative therapy for pregnant women, and malaria case diagnosis and treatment with artemisinin-based combination therapy (ii) larval source management (LSM- consisted of draining, filling and larviciding) + house improvement	(i) House improvement alone, (ii) LSM alone	36 months	Entomological inoculation rate (EIR), Mosquito density, *P. falciparum* prevalence	The mean nightly EIR fell from 0.010 infectious bites per person (95% CI 0.006–0.015) in the baseline year to 0.001 (95% CI 0.000–0.003) in the last year of the trial. Over the full trial period, the EIR did not differ between the four trial arms (p = 0.33). Similar results were observed for the other outcomes: mosquito density and P. falciparum prevalence decreased over 3 years of sampling, while haemoglobin levels increased; and there were minimal differences between the trial arms during the trial period. -In the context of high insecticide-treated bed net use, neither community-based house improvement, LSM, nor house improvement + LSM contributed to further reductions in malaria transmission or prevalence beyond the reductions observed over two years across all four trial arms
Furnival-Adams et al. [61]	Côte d’Ivoire	Experimental study design	To evaluate the efficacy of indoor ATSB® traps treated with 4% boric acid (BA ATSB) or 1% chlorfenapyr (CFP ATSB) in combination with untreated nets or LLINs (holed or intact), against pyrethroid resistant *Anopheles gambiae *sensu lato	General population	Arm 1- Boric acid 4% ATSB + untreated polyester net holed Arm 2- Boric acid 4% ATSB + PermaNet 2.0 LN holed Arm 3- Chlorfenapyr 1% ATSB + untreated polyester net holed. Arm 4- Chlorfenapyr 1% ATSB + PermaNet 2.0 LN holed Arm 5- Chlorfenapyr 1% ATSB + PermaNet 2.0 LN intact	Arm 6-Untreated polyester net holed Arm 7-PermaNet 2.0 LN holed	4 months	Mosquito mortality	The addition of ATSB to LLINs increased the mortality rates of wild pyrethroid-resistant An. gambiae from 19% with LLIN alone to 28% with added BA ATSB and to 39% with added CFP ATSB (p < 0.001). Anopheles gambiae mortality with combined ATSB and untreated net was similar to that of combined ATSB and LLIN regardless of which insecticide was used in the ATSB. The presence of holes in the LLIN did not significantly affect ATSB-induced An. Gambiae mortality. Comparative tests against pyrethroid resistant and susceptible strains using oral application of ATSB treated with pyrethroid demonstrated 66% higher survival rate among pyrethroid-resistant mosquitoes
Deparis et al. [62]	Coˆ te d’Ivoire	Experimental study design	To assess the field efficacy of the impregnated battlefield uniforms (BFUs) and their resistance to washing	French military personnel	Impregnated BFU + 50% DEET Topical repellent	Impregnated BFU alone, or DEET alone	2 months	Malaria incidence, protective effects of DEET	The protective effects of the use of DEET skin repellent was not significant, perhaps due to the high density of Anopheles mosquitoes during the night catching sessions and an average time of effective repellency of 2 or 3 h in the field. The analysis indicated that the industrial impregnation of permethrin of the BFU offered some protection from mosquito bites but not enough to reduce significantly the incidence of malaria among nonimmune troops. No positive or negative interaction was noted when DEET and the impregnated BFUs were used together
Allcock et al. [63]	Namibia	A cross-sectional study	To explore the coverage of two vector control methods: IRS and ITNs	2–10 years	IRS + ITN	Single intervention data	Not clear	PfPR. PfPR 2–10 (the proportion of the population aged 2–10 years carrying asexual blood parasites)	PfPR2–10 ≥ 5% was strongly associated with IRS (RR 14.54; 95% CI 5.56–38.02; p < 0.001), ITN ownership (RR 5.70; 95% CI 2.84–11.45; p < 0.001) and ITN and/or IRS coverage (RR 5.32; 95% CI 3.09–9.16; p < 0.001). Transmission intensity was strongly associated with intervention coverage, with households in the PfPR2 –10 ≥ 5% category the most likely to have at least one intervention (RR 6.10; 95% CI 3.74–9.97; p < 0.001)
Rojas et al. [64]	Colombia	A cross-sectional study	To implement and evaluate an Integrated Malaria Control Program	General population	Drainage filling + bromelias removal	None	36 months	Malaria deaths, cases of cerebral malaria, malaria incidence, and length of sick leave	The project: (1) avoided deaths from malaria (no fatal cases in the 3-year period, compared to 5–8 deaths a year previously); (2) avoided cases of cerebral malaria (no cases, as compared to 90–110 per year previously); (3) reduced malaria incidence by 45.36%; (4) decreased length of sick leave from 7.52 to 3.7 days
Chen-Hussey et al. [65]	Lao People’s Democratic Republic	A double blind, household randomised, placebo-controlled trial	To determine whether the use of repellent and LLINs could reduce malaria more than LLINs alone	6–60 years	LLIN + DEET lotion	Placebo lotion	11 months	Malaria incidence	Intention to treat analysis found no effect from the use of repellent on malaria incidence (hazard ratio: 1.00, 95% CI 0.99–1.01, p = 0.868)
Pinder et al. [66]	Gambia	Two-arm cluster, randomised, controlled efficacy trial	To assess whether the addition of IRS to LLINs provided a significantly different level of protection against clinical malaria in children or against house entry by vector mosquitoes	Children, aged 6 months to 14 years	LLIN + IRS	LLIN alone	24 months	Incidence of clinical malaria, density of vector mosquitoes	Incidence of clinical malaria was 0·047 per child-month at risk in the LLIN group and 0·044 per child-month at risk in the IRS plus LLIN group in 2010, and 0·032 per child-month at risk in the LLIN group and 0·034 per child-month at risk in the IRS plus LLIN group in 2011. The incident rate ratio was 1·08 (95% CI 0·80–1·46) controlling for confounders and cluster by mixed-effect negative binomial regression on all malaria attacks for both years. No significant difference was recorded in the density of vector mosquitoes caught in light traps in houses over the two transmission seasons: the mean number of *A. gambiae sensulato* mosquitoes per trap per night was 6·7 (4·0–10·1) in the LLIN group and 4·5 (2·4–7·4) in the IRS plus LLIN group (p = 0·281 in the random-effects linear regression model)
Sluydts et al. [67]	Cambodia	A cluster randomised controlled trial	To assess the epidemiological efficacy of a highly effective topical repellent in addition to long-lasting insecticidal nets in reducing malaria prevalence in this setting	General population	LLIN + topical repellent	LLIN alone	24 months	Plasmodium species prevalence	No post-intervention differences in PCR plasmodium prevalence were observed between study groups in 2012 (4·91% in the control group vs 4·86% in the intervention group; OR 1·01 [95% CI 0·60–1·70]; p = 0·975) or in 2013 (2·96% in the control group vs 3·85% in the intervention group; OR 1·31 [0·81–2·11]; p = 0·266). Similar results were obtained according to Plasmodium species [Plasmodium falciparum; OR 0·83 [0·44–1·56]; p = 0·561; and Plasmodium vivax; OR 1·51 [0·88–2·57]; p = 0·133)]. 41 adverse event notifications from nine villages were received, of which 33 were classified as adverse reactions (11 of these 33 were cases of repellent abuse through oral ingestion, either accidental or not)
Martins-Campos et al. [68]	Brazil	Cross-sectional study	To investigate the fauna of anopheline mosquitoes and verify the impact of integrated vector management in two colonization projects in the Careiro Municipality, Western Brazilian Amazon	General population	ITNs + IRS	ITNs alone	20 months	HBRs, EIRs, malaria incidence rate, plasmodium carrier’s prevalence	*An. darlingi* HBRs showed a notable decreasing trend from the start to the end of the study. Conversely, *An. albitarsis* increased its contribution to overall HBRs throughout the study. For *An. darlingi* there was a significant positive correlation between HBRs and malaria incidence rate (p = 0.002). *Anopheles albitarsis* HBRs showed a significant negative correlation with the corresponding malaria incidence rate (p = 0.045). EIR from total anophelines and from *An. darlingi* and *An. albitarsis* presented decreasing patterns in the successive collections. Four species of anophelines (*An. darlingi, An. albitarsis, An. braziliensis and An. nuneztovari*) were naturally infected with Plasmodium, albeit at very low infection rates. There was a decrease in the malaria incidence rate for both vivax and falciparum malaria and in the prevalence of *Plasmodium vivax* and *Plasmodium falciparum* carriers during the period of study
Hill et al. [69]	Bolivia	A double blind, placebo-controlled, cluster-randomised clinical study	To determine the effectiveness in reducing malaria of combining an insect repellent with insecticide treated bed nets compared with the nets alone in an area where vector mosquitoes feed in the early evening	General population	LLINs + plant-based insect repellent	Placebo	7 months	Episodes of *Plasmodium falciparum* or *P. vivax* malaria, Numbers of *P. falciparum* cases	Analysed 15 174 person months at risk and found a highly significant 80% reduction in episodes of *P. vivax* in the group that used treated nets and repellent (incidence rate ratio 0.20, 95% CI 0.11 to 0.38, P < 0.001). Numbers of P falciparum cases during the study were small and, after adjustment for age, an 82% protective effect was observed, although not significant (0.18, 0.02 to 1.40, P = 0.10). Reported episodes of fever with any cause were reduced by 58% in the group that used repellent (0.42, 0.31 to 0.56, P < 0.001)
Kane et al. [70]	Mali	A comparative study, cross-sectional and passive case detection surveys	To assess the added value of IRS to LLINs on the prevalence of parasitaemia and malaria incidence among children under 10 years old	Children from 6 months to 10 years old	IRS + LLINs	LLINs only	Not clear	Malaria prevalence and incidence	There was an increase of 220% in malaria prevalence from June to October in the control area (14% to 42%) versus only 53% in the IRS area (9.2% to 13.2%). Thus, the proportional rise in malaria prevalence from the dry to the rainy season in 2016 was 4-times greater in the control area compared to the IRS area. The overall malaria incidence rate was 2.7 per 100 person-months in the IRS area compared with 6.8 per 100 person-month in the control areas. The Log-rank test of Kaplan–Meier survival analysis showed that children living in IRS area remain much longer free from malaria (Hazard ratio (HR) = 0.45, 95% CI 0.37–0.54) than children of the control area (P < 0.0001)
Lee et al. [71]	Island of Príncipe	Cross- sectional survey, before and after study	To evaluate a five-year integrated control programme	General population	IRS + LLINs + IPT for pregnant women + early diagnosis and prompt treatment with artemisinin-based combination therapy	IRS alone, LLIN alone	60 months	Protective effect against malaria, malaria incidence and prevalence, malaria mortality, slide positivity rate was used as an indicator of any increase of malaria cases during and after the control programme	Combined use of IRS and LLINs has no additional protective effect against malaria when compared to the use of IRS alone (OR = 1.108, 95% CI 0.594–2.066, p-value = 0.747 > 0.05). Being unprotected increases the odds by 3.5 (OR = 3.496, 95% CI: 1.473–8.300, p-value = 0.005) of that for IRS protection alone, while using LLINs alone, when compared with IRS protection alone, increased the odds by almost 3-times (OR = 2.979, 95% CI 1.113–7.975, p-value = 0.030). Meaning that there is no statistical evidence that living in an IRS treated house with a bed net has any additional protective effect against malaria infection - A steep decline by 99% of malaria incidence was observed between 2003 and 2008, with an incidence risk of the population of five per thousand, in 2008. No malaria mortality reported since 2005. Species shift from falciparum to non-falciparum malaria was noted after a five-year intensive control programme - Before the intervention was taken, higher incidence of clinical and severe malaria was noted in under-five years of age children and a reduction (from 40 to 20%) observed
Mathews et al. [72]	Cameroon	Cross- sectional study. Pre-and Post-intervention medical survey	To examine different mosquito control interventions applied to entire villages to assess their impact on vectors, malaria incidence and the quality of life of the communities	Children and adults	IRS + ITN, OR Improved screening of houses + outdoor/intradomiciliary misting	A village received no treatment	8 months	Malaria incidence, numbers of mosquitoes	IRS + ITN using ICON CS (lambda-cyhalothrin capsule suspension formulation) or improved screening of houses combined with outdoor misting reduced the numbers of mosquitoes collected from exit traps compared to the other treatments. More sporozoites were detected in mosquitoes sampled in exit traps in the untreated village than in the treated villages. Malaria incidence several months after treatments was not significantly different from pre-treatment levels. Blackfly adult populations were reduced for several weeks following larvicide application but recovered when treatment was halted
Protopopoff et al. [73]	Burundi	Cross-sectional surveys	To present the impact of these targeted vector control activities on the prevalence of malaria infection	Children between 1- 9 years	IRS + LLIN	LLIN alone	48 months	Prevalence of malaria, parasite density, risk of malaria infection, histories of malaria illnesses and antimalarial drug use	After the intervention and compared with the control valleys, children 1–9 years old in the treated valleys had lower risks of malaria infection (OR: 0.55), high parasite density (OR: 0.48), and clinical malaria (OR: 0.57). The impact on malaria prevalence was even higher in infants (OR: 0.14). When intervention with control valleys were compared, children of age 1–9 years had a significantly lower risks of malaria infection [OR: 0.55, 95% CI 0.42–0.72, P < 0.001], high-density parasitemias (OR: 0.48, 95% CI 0.33–0.70, P < 0.001), and clinical malaria (OR: 0.57, 95% CI 0.41–0.81, P = 0.001). Histories of malaria illness (OR: 0.66, 95% CI 0.52–0.83, P < 0.001) and antimalarial drug use (OR: 0.65, 95% CI 0.49–0.85, P = 0.002) were lower in the intervention valleys compared with the control valleys. According to surveys, malaria prevalence was reduced in intervention valleys compared with control valleys by 12–64% in the ≤ 9 age group and by 14–59% in > 9 age group. These differences were significant in children ≤ 9 years old for surveys 3, 5, and 9 and in individuals > 9 years old for Surveys 3–6 and 9. No difference in malaria prevalence was observed between intervention hilltops and control hilltops, where using nets did not confer an additional protective effect to spraying. Targeted vector control had a major impact on malaria in the high-risk valleys but not in the less-exposed hilltops
Hiwat et al. [74]	South America	Cross-sectional study, before and after evaluation	To evaluate both on account of the targets established within the programme and on account of its impact on the malaria situation in Suriname	General population	Interventions grouped by strategic areas: vector control (including IRS, LLNs, re-/impregnation of nets and entomological surveillance); case management (diagnosis and treatment); Behaviour Change Communication/Information, Education and Communication (mass media, outreach programme) and Surveillance, Monitoring and Evaluation (including epidemic detection, passive and active case surveillance, mobile/fixed malaria service deliverers, M&E)	None, before and after data	60 months	Malaria vector populations, malaria incidence and transmission	Malaria vector populations, monitored in sentinel sites, collapsed after 2006 and concurrently the number of national malaria cases decreased from 8618 in 2005 to 1509 in 2009. Malaria transmission risk shifted from the stabile village communities to the mobile gold mining communities, especially those along the French Guiana border
Bradley et al. [75]	Equitorial Guinea	Cross-sectional study	To examine the effect of the short residual life of bendiocarb insecticide and of children spending time outdoors at night, on malaria infection prevalence was	Children 2–14 years	ITN + IRS	ITN	6 months	Prevalence of malaria infection	Prevalence of malaria infection in 2 to 14-year-olds in 2011 was 18.4%, 21.0% and 28.1% in communities with median time since IRS of three, 4 and 5 months respectively. After adjusting for confounders, each extra month since IRS corresponded to an OR of 1.44 (95% CI 1.15–1.81) for infection prevalence in 2 to 14-year-olds. Mosquito mortality was 100%, 96%, 81% and 78%, at month 2, 3, 4 and 5 respectively after spraying. Only 4.1% of children spent time outside the night before the survey between the hours of 22.00 and 06.00 and those who did were not at a higher risk of infection (OR 0.87, 95% CI 0.50–1.54). Sleeping under a mosquito net provided additive protection (OR 0.68, 95% CI 0.54–0.86)

### Quality or risk of bias assessment of individual studies

Two reviewers (EA and CN) independently assessed and scored the quality of selected studies. Observational studies were assessed according to the Newcastle–Ottawa scale (NOS) [19] where studies are scored between zero to nine stars for nine questions that cover three areas: selection, comparability and outcome. For randomized controlled trials (RCTs), Cochrane Collaboration’s tool (RoB tool) for assessing the risk of bias in randomized trials [20] was used. This tool provides judgment whether the study is having high, moderate, low or unclear risk of bias. Inconsistencies in the findings were resolved by discussion (EA and CN reaching consensus or by involving a third reviewer (RN or DM) where necessary).

### Data synthesis and analysis

Review data was synthesized narratively while answering the review question (does use of two or more malaria prevention methods holistically at households or in the community lead to reduced occurrence of the disease (primary outcome) or presence of mosquitoes in houses (secondary outcome). Findings are descriptively presented and discussed while elaborating integrated malaria interventions and the related primary and secondary outcomes. Data are presented in tabular form for comparison, highlighting country, year of study, study objective, intervention, context, population and outcomes among others.

## Results

In total, 10,931 records were identified by the search strategy from databases (n = 6652 studies) and grey literature (n = 4279). After screening titles (removing duplicates and irrelevant information), 137 studies were retained and their abstracts screened. A total of 74 articles were accessed and screened at full text level. Of these, 17 were excluded for different reasons such as not addressing the review objective, as well as being mathematical models or cost-effectiveness analyses, leading to 57 articles which were used in the review. A flow chart with details of the article screening process is shown in Fig. 1.

### Characteristics of included studies

#### Study setting

Of the included 57 studies, majority (n = 10) were conducted in Tanzania [21–30]; followed by Kenya (n = 7) [10, 31–36]; Ethiopia (n = 5) [11, 32, 37–39] including one study in both Kenya and Ethiopia [32]; and Uganda (n = 5) [40–44]. Mozambique [45–47], Benin [48–50], Ghana [51–53], and India [54–56] had 3 studies each. Two studies were from Nigeria [57, 58], Malawi [59, 60]; and Cote d’Ivoire [61, 62]. Other articles were from Namibia [63]; Colombia [64]; Lao People’s Democratic Republic [65]; The Gambia [66]; Cambodia [67]; Brazil [68]; Bolivia [69]; Mali [70]; Island of Príncipe [71]; Cameroon [72]; Burundi [73]; Suriname [74]; and Equatorial Guinea [75].

#### Risk of bias

Overall, the quality of studies included in the review was generally fairly good. Most RCTs (n = 11) were of moderate risk [21, 22, 24, 27, 32, 35, 38, 49, 60, 65]; while three were of low risk [39, 66, 69] and two of high risk [47, 67]. Although several pre-post evaluation studies were scored as good quality [10, 33, 34, 41, 42, 45, 59, 68, 70–74], others had fair scores [11, 29, 40, 44, 48, 54–56, 75]. Only one quasi-experimental study was rated good [25], while others fair [23, 26, 30, 31, 50, 61, 62]. The cross-sectional surveys included [36, 37, 43, 46, 51–53, 57, 63] were generally rated good quality and a few (n = 2) [36, 37] were rated fair. Given the nature of the interventions, blinding of participants and evaluators was generally absent in most studies. Some information including details of intervention concealment, response rate, and use of validated measures were not clearly provided which limited objective judgement of the quality of some studies.

#### Study population

The studies involved different community-based interventions on malaria control that targeted general populations [10, 11, 22, 23, 25–37, 39–64, 66–68, 70, 71, 73–75], including those that focused on residents in controlled intervention households [21, 24, 38, 65, 69, 72], pregnant women and children [51, 58], and children care takers [57]. A few studies involved individuals of all ages [65, 72], household members older than 6 months [21, 24], while several studies reported on malaria outcomes in children of different ages including: below 6 years [34, 46, 47, 49]; 0.5–14 years [27, 28, 66]; 0.5–10 years [41, 42, 70], 2–10 years [63], 0.5–13 years [33], under 10 years [59], 2–14 years [75], 1–15 years [58], and primary school children [29, 32]. One study [62] was among the French Military troops in Côte d’Ivoire. The studies using experimental designs generally used adult volunteers.

#### Malaria prevention interventions

Various interventions were used, which were mostly combinations of two or three malaria prevention methods including ITN, IRS, topical repellents, insecticide sprays, microbial larvicides, and house improvements including screening, wall hangings and eaves screening. Of the intervention studies, majority (n = 22) assessed ‘ITN plus IRS’ [9, 10, 23, 27, 28, 34, 35, 39, 41, 44, 47, 48, 50, 66, 70–77], followed by the ‘ITN plus topical or spatial repellents’ (n = 9) [21, 24, 38, 54, 55, 59, 65, 67, 69]. Larval source management was another intervention used in different combinations including with: (a) ITNs [33]; (b) larviciding, LLINs, IRS, and mass drug administration [50]; (c) IRS, LLINs, intermittent preventive treatment for pregnant women, as well as early diagnosis and prompt treatment with artemisinin-based combination therapy, and larviciding [71]; (d) chemical larviciding, IRS, space spraying of insecticides at ultra-low volume, ITNs, and environmental management [29]; (e) LLINs, screening in windows and ventilators, removing mosquito breeding, and early closing of doors [40]; (f) LLINs plus larviciding with *Bacillus thuringiensis* var. israelensis (Bti) and community education and mobilization [32]; and (g) housing improvement [60]. Besides intervention studies, the review also included some cross-sectional surveys that analysed data regarding different malaria control combinations such as ITNs and IRS [37, 43, 46, 51, 53, 63]; ITNs and household insecticide products such as spatial repellent, smoke, and insecticide canister sprays [36, 52]; and ITNs, screening of windows, regular environmental sanitation, and insecticide sprays [57].

#### Study designs

The studies included cluster RCTs (n = 16) [21, 22, 24, 27, 28, 32, 35, 38, 39, 47, 49, 60, 65–67, 69], and evaluation of programmes/interventions (n = 17) [11, 29, 34, 40, 44, 45, 48, 54–56, 59, 70–75]. Other studies included national Demographic and Health Survey [63]; panel data from administrative districts [52]; hospital data [43, 51]; and other surveys [36, 37, 46, 53, 57, 58]. Experimental hut/houses and field trials [23, 25, 26, 30, 31, 50, 61, 62, 68] were also included.

### Primary outcomes

#### Malaria incidence

A total of 24 studies reported malaria incidence alone [10, 21, 33, 35, 39, 41–43, 45, 47–49, 54, 63–66, 68–74], with most (14) indicating significantly reduced incidence in integrated interventions compared to single ones [10, 33, 41, 43, 47, 48, 54, 63, 64, 68–70, 73, 74]. Most studies used IRS plus ITN combinations [10, 41, 43, 47, 48, 63, 70], some of which were regarded as cost-effective in urban areas [48], while others significantly reduced malaria in all-age groups (61%) [10] or among children aged 2–10 years in high transmission areas [47, 63, 70]. Two studies using IRS plus ITNs showed that non-pyrethroid IRS, such as pirimiphos-methyl was more effective than pyrethroids for IRS in areas with widespread pyrethroid resistance (incidence rate of 2.7 per 100 person-months in the intervention area compared with 6.8 per 100 person-month in the control area) [70] and in highly endemic areas (incidence rate of 3532 per 10,000 children-month in intervention group versus incidence rate of 4297 per 10,000 children-month in the control group [47]. A combination of several interventions including filling ditches, larval source management, education, as well as strengthened diagnosis and treatment mechanisms reduced cerebral malaria (from 90 to 110 to zero cases annually); and malaria incidence by 45% [64]. A combination of nets with a plant-based insect topical repellent significantly reduced episodes of fever including 80% reduction in *Plasmodium vivax* episodes in comparison to nets alone [69], and microbial larvicides plus ITNs reduced the risk of acquiring new infection in 1.5–13 year olds in comparison to ITNs alone [33].

However, no significant incidence reductions in the integrated approach were reported in 10 studies [21, 35, 39, 42, 45, 49, 65, 66, 71, 72] including nets plus topical repellent [21, 65]; LLINs plus IRS or carbamate-treated plastic sheeting plus LLINs combination [49]. Weekly larviciding of stagnant water bodies combined with nets, IRS, and mass drug administration showed modest reduction in human infections [35]. In another study, a significant reduction in all-age incidence was recorded, and over 76.7% of expected cases were averted. However, effects on malaria prevalence were varied and not associated with expected level or trend changes in an area with significant pyrethroid resistance [45].

#### Malaria prevalence

Of 22 studies [11, 27–29, 32, 34, 36–38, 40, 46, 51–53, 56–60, 67, 73, 75, 76] reporting prevalence alone, most (15) showed more benefits in the integrated approach [11, 28, 29, 36–38, 40, 46, 51–53, 56, 57, 59, 73]. These included combination of daily application of topical repellents during the evening plus use of LLINs at community level [38]; as well as spatial repellent devices plus LLINs in endemic villages [59]. Compared to single or no intervention at all, IRS plus LLIN combinations [28, 37, 46, 73, 75] specifically reduced malaria in all ages [37] and children between 0.5 and 14 years [28, 46, 73, 75] including across a range of transmission intensities and net utilization levels (76). Some large-scale multiple intervention combinations of IRS plus ITNs plus behaviour change/communication strategies reduced prevalence with greater impact in high-risk areas [73], as well as among females [52] and children under 5 years [51–53]. Prevalence reductions in children under 5 years were also related to LLINs mass campaigns alongside other anti-malarial interventions which reduced malaria cases (by 50%) and deaths (by 65%) [51]. Other combinations of multiple interventions including community-based education promoting integrated vector management plus environmental management, larviciding plus LLINs and IRS [11]; early detection and prompt treatment plus larvivorous fishes [56]; and vector control, rapid diagnosis and treatment, and community health education [29] also significantly reduced prevalence in comparison with single methods.

However, mixed (n = 1) [75] and non-significant effects (n = 6) [27, 32, 34, 58, 60, 67] of integrated approaches on malaria prevalence were also reported. For instance, adding ITNs to IRS had a significant impact at baseline and immediately after the first round of IRS, but showed limited effects after the second round when the IRS impact was strongest [34]. Similar mixed results were noted among primary school children where combinations of three interventions (LLINs plus *Bti* plus community education and mobilization arm, compared to the LLINs only arm, LLINs plus Bti arm, and LLINs plus community education and mobilization arm) were used in a low prevalence setting, but not at sites with relatively higher prevalence [32]. In another study, there was no significant contribution of community-based house improvement and/or larval source management to reductions in malaria prevalence beyond the reductions provided by the mass ITN distribution, community engagement programme and other national malaria control interventions [60]. Furthermore, a topical mosquito repellent (picaridin) plus LLINs versus LLINs group alone showed no significant differences in *Plasmodium* prevalence [67], similar to results of insecticide sprays plus ITNs in another study [58]. One study [75] showed mixed effects of LLINs and IRS combination, with a rapid decline of IRS insecticidal effectiveness 3 months following spraying posing considerable operational concerns given that malaria transmission occurred throughout the year.

### Secondary outcomes

#### Human biting and entomological inoculation rates

Human biting rates (HBRs) [30, 41, 44, 68], and entomological inoculation rates (EIRs) [22, 44, 60, 68], were significantly reduced in the integrated approach compared to single interventions. For example, ITNs plus IRS combinations were associated with a significant decrease in: *Anopheles darlingi* HBRs trend; EIR from total anophelines; *Anopheles darlingi* and *Anopheles albitarsis* [68]; HBR of female *Anopheles* mosquitoes [41, 44]; and annual EIRs decline [44]. Similarly, a study of carbamate IRS plus ITNs produced major reduction in EIRs compared to ITNs alone in an area of moderate coverage of LLINs and high pyrethroid resistance in *Anopheles gambiae *sensu stricto [22]. Compared to LLINs with untreated baskets [30], transfluthrin-treated baskets combined with LLINs reduced the proportion of *An. arabiensis* mosquito bites by more than three quarters, and *Anopheles funestus *sensu lato* (s.l.)* mosquitoes bites by nearly half [30]. However in another study, reductions in EIR over the full trial period did not significantly differ between the four trial arms (control, house improvement, larval source management, nor house improvement and larval source management) [60].

#### Mosquito deterrence and mortality

Mosquito deterrence (related to preventing mosquito entry into houses) [23, 30, 50] and mosquito mortality [23, 25, 30, 50, 61] were also evaluated in some studies, indicating benefits of using a combination of methods compared to single ones. For example, sisal decorative baskets treated with transfluthrin repellents induced a tenfold increase in 24-h mortality of *Anopheles arabiensis* mosquitoes, providing additional household and personal protection against indoor biting malaria and nuisance mosquitoes in the early evening [30]. This combination intervention also deterred three-quarters of *An. arabiensis* mosquitoes from entering huts in comparison with untreated nets and IRS alone [30]. In one study, indoor mosquito traps in combination with LLINs enhanced mortality of pyrethroid-resistant *An. gambiae* compared to single interventions [61]. In another study, use of chlorfenapyr and alpha-cypermethrin together as a mixture on nets or a combined chlorfenapyr IRS and pyrethroid LLIN intervention provided better deterrence of *An. gambiae s.l.* and induced significantly higher levels of mortality of pyrethroid-resistant malaria vectors [50].

#### Mosquito densities

Mosquito densities [22, 24, 32, 35, 37, 55, 60, 66] generally reduced significantly in the combined approach than the reference groups [22, 24, 26, 37, 55]. The ITN plus IRS arm in one study was associated with significant reduction in overall mosquito density [37], and mean *An. gambiae s.l.* density [22] compared to control groups. In another study, LLINs in combination with topical repellents where everybody received 15% N,N-Diethyl-meta-toluamide (DEET) had resting mosquito densities fewer than half that of households in the placebo scenario [24]. Similarly, the total anopheline density, including *Anopheles dirus*, *Anopheles minimus* and *Anopheles philippinensis*, in houses in mosquito nets plus topical repellent arm declined in comparison with other arms (ITNs, topical repellent, and no treatment) [55]. A series of preliminary experiments evaluating eave tubes indicated that installing them plus screening following introduction of LLINs in a model village reduced larval density greatly compared to pre intervention values, and virtually eliminated indoor host-seeking mosquitoes [26].

On the other hand, there were no significant differences in mosquito density in some studies [32, 35, 60, 66]. Specifically, 3 years of sampling in trial arms (house improvement, larval source management, house improvement and larval source management) [60], and combining LLINs with larviciding with Bti plus community education and mobilization showed no significant differences in reduction of adult anopheline density in each of the groups [32]. In addition, despite the high coverage of interventions targeting malaria hotspots, no statistically significant difference was observed in the mosquito densities of female anophelines between the intervention (larviciding, LLINs plus IRS plus focal mass drug administration) arms and control clusters (standard national programme) [35].

## Discussion

This systematic review synthesized evidence on integrated malaria prevention and its effectiveness in controlling the disease in LMICs. The study found various combinations of prevention methods used in malaria control across countries, with ITNs and IRS the most utilized, followed by various combinations including topical repellents, environmental management, and larviciding. The use of several malaria prevention measures in combination was effective in reducing both malaria incidence and prevalence in several studies in comparison with single methods. The review also indicated a reduction in human biting and entomological inoculation rates and mosquito densities, as well as an increase in mosquito deterrence and mortality following implementation of a combination of malaria prevention methods. The improved malaria outcomes related to occurrence of the disease and mosquito abundance in the studies can be related to the synergistic effect of combining several methods to prevent the disease. However, some studies found no additional benefits of using combinations of several malaria prevention measures in comparison with single interventions. Overall, these findings from the systematic review show promise in the use of multiple malaria prevention methods holistically to complement existing strategies in endemic countries striving for malaria elimination [1]. Such evidence is needed to contribute to the WHO global vector control response particularly for malaria, a leading cause of morbidity and mortality in many LMICs [77].

The review found that except in a few studies where the effect was modest or mixed [34, 49], ITNs and IRS combinations reported reduced malaria incidence and prevalence [10, 28, 37, 41, 43, 46–48, 63, 70, 73, 75]. Indeed, a systematic review on the effect of adding IRS to communities using ITNs established reduced prevalence of malaria [78]. When ITNs and IRS are used in combination, their synergistic effect is likely to be greater as ITNs would be most effective against vectors that primarily feed late at night while IRS would be most effective against vectors that spend much of their adult lives resting inside houses [79]. Indeed, these two methods are most effective in areas where predominant mosquito vectors are both strongly endophagic and endophilic [34]. Other research has demonstrated that the combined effect of ITNs and IRS could depend on the levels of malaria transmission in the area. A nationally representative survey from 17 sub-Saharan African countries using the two methods indicated that intervention effects varied across malaria transmission levels. Indeed, ITNs were associated with a significant reduction in malaria morbidity in high and medium transmission settings, while IRS appeared to be most effective in medium and low transmission areas [9]. In a related review, integrated malaria prevention using ITNs and IRS contributed to a reduction in malaria incidence but had little impact on prevalence dependent on transmission levels [80]. In this study, the use of both interventions together showed more protection than each intervention on its own especially in medium transmission settings. Therefore, whereas existing evidence predominantly demonstrates additional benefits of combining ITNs and IRS, malaria transmission levels in the target areas need to be considered while planning such interventions. In addition, optimal coverage of either ITNs or IRS should be prioritized before introduction of another malaria prevention intervention. Indeed, the WHO has discouraged the use of the second intervention to compensate for deficiencies in the first malaria control method [81].

Beyond use of ITNs and IRS, the systematic review shows reduced incidence and prevalence of malaria while using a combination of other methods. There was reduced malaria incidence/prevalence while using the following combinations: environmental management, health education and case management [64]; ITNs and repellents (topical and spatial) [38, 59, 69]; larviciding and ITNs [33]; health education and environmental management [11]; and case management and health education [29]. However, many of these methods such as larviciding and environmental management, that have been known to reduce breeding of mosquitoes for many years, have been largely ignored in many endemic communities [82]. Indeed, the use of other malaria prevention methods in many LMICs beyond ITNs and IRS is minimal despite the WHO recommending the use of a mix of chemical and non-chemical measures to prevent the occurrence of the disease [83]. The non-core malaria prevention methods should be explored to complement existing strategies. Such approaches could offer significant benefits despite related challenges such as high cost of repellents [84]; environmental management being cumbersome [85]; and larviciding being labour intensive and expensive [86]. In promoting integrated malaria prevention beyond ITNs and IRS, barriers and facilitators of use of the different measures in LMICs should be considered. In addition, the current evidence for each of the malaria prevention methods should inform their use in particular settings. For example, larviciding has been recommended to supplement ITNs and IRS in areas where aquatic habitats are few, fixed and findable, whereas more robust research is needed on environmental management as a strategy for malaria control [81].

The review findings show that use of some multiple malaria prevention methods led to reduced human biting [30, 41, 44, 68] and entomological inoculation rates [22, 44, 60, 68], as well as an increase in mosquito deterrence [23, 30, 50] and mosquito mortality [23, 25, 30, 50, 61] in comparison with single interventions. These findings could partly explain the mechanism of reduced malaria occurrence observed in this systematic review. Indeed, reduced presence and biting of mosquitoes indoors for example due to improved housing is directly related to low malaria transmission [87]. In addition, some of the methods predominantly used in the studies included in the review such as ITNs provide protection against biting from mosquitoes [81]. Whereas some malaria prevention methods such as ITNs and IRS are used indoors, many of the other interventions that were being used in the integrated approach such as environmental management, larviciding and housing improvement target mosquito populations before entering houses. Therefore, these methods directly contribute to lower numbers of mosquitoes indoors which could result in reduction in the occurrence of malaria. Although ITNs protect users while sleeping, evidence suggests that mosquitoes may bite hence transmit malaria before one goes to bed or outdoors in many endemic countries [88, 89]. This occurrence emphasizes the need to explore interventions that not only target mosquitoes indoors but also those that reduce mosquito breeding and prevent their entry into houses as advocated in integrated malaria prevention. It is also important to note that effectiveness of ITNs and IRS is dependent on the ability of mosquito vectors coming into contact with the insecticides, as well as susceptibility to the insecticides used. Overall, the varied effects in different studies observed in the review demonstrate that appropriate combinations that are not “one size fits all” should be recommended in consideration of the complex and dynamic nature of mosquito populations, insecticide resistance patterns, local epidemiology, and the operational effectiveness of malaria control interventions [9, 41].

Some studies in the systematic review showed mixed results or no benefits of using multiple approaches to prevent malaria at households and in communities [21, 27, 32, 34, 35, 39, 42, 45, 49, 58, 60, 65–67, 71, 72, 75]. Although there were no observed differences in these studies compared to those that demonstrated benefits of using multiple methods, several factors could potentially explain these findings from the review. First of all, the effects of certain malaria prevention methods such as ITNs and IRS which dominated in the various studies are likely influenced by the behaviour and resistance status of the primary malaria vectors. Indeed, mosquitoes could develop resistance to certain insecticides leading to reduced efficacy of deployed methods [82, 83]. In addition, while innovative mixes of interventions could achieve large reductions in disease burden [45], there are concerns about the short residual life of the insecticides used in IRS and the related need for additional rounds of spraying. These concerns could pose cost-effectiveness issues, excessive demand on the spray programme, and households’ non-compliance with re-spraying of their houses [75]. Acceptability and efficacy challenges have also been reported regarding IRS use in some communities [84] hence could explain some of the non-significant results.

It is also worth noting that there is need for more evidence on the effectiveness of some of the methods in malaria prevention such as spatial repellents, larvivorous fish, and larval habitat modification as recommended by the WHO [81]. Future studies on individual and combinations of various prevention methods (exploring possible synergistic effects) are needed to add to the much needed literature on malaria control beyond common interventions such as ITNs and IRS. Such research should be context specific, considering how various attributes such as mosquito density and behaviour, existing evidence on proposed designs, and coverage of other malaria prevention approaches impact the mosquito or disease related outcomes. For instance, some studies highlight the importance of net coverage in determining the effect of the IRS plus ITNs combination [27], and that high LLIN coverage is sufficient to protect people against malaria in areas of low or moderate transmission. Findings indicate that where ITN coverage is low, additional control with IRS could be needed [49, 66] as IRS is considered a secondary measure, and only crucial when ITNs have not been effective [23]. Indeed, in settings where ITN coverage is optimal, the addition of IRS may add minimal benefit in reducing malaria morbidity and mortality [81]. There are also concerns regarding the fidelity of some interventions in the integrated approach such as environmental management being cumbersome [85] which could also have influenced the findings. More evidence is therefore needed to further explain why combinations of certain malaria prevention methods may not be as effective in certain studies and contexts.

A limitation of this review is that outcomes in the included studies were measured differently which may have affected the results. This review also considered only articles published in English which could have led to publication bias. In addition, having included studies from only LMICs could have omitted interesting findings from other countries. Furthermore, there were few RCTs and many studies were generally of fair quality which can impact the level of evidence obtained. Nevertheless, this review is the first to synthesize evidence on integrated malaria prevention which should contribute to malaria control efforts in endemic countries. The 20-year period used in the review provided a sufficient duration to include not only recent studies but also evidence on combinations of various malaria prevention methods used many years ago. However, it is recognized that there could have been changes in malaria control recommendations and guidelines during this period which could have influenced the quality and extent of interventions used at various times and resultant findings.

## Conclusions

Use of multiple malaria prevention methods was effective in reducing malaria incidence, prevalence, human biting and entomological inoculation rates, as well as increasing mosquito deterrence and mortality in comparison with single methods. However, a few studies showed mixed results or no benefits on using multiple approaches to prevent malaria. More evidence is needed on the effectiveness of some malaria prevention methods for malaria control used individually or in combination. Results from this systematic review could inform future research, as well as practice, policy and programming on integrated malaria prevention in endemic countries to add to existing national and global control efforts.

## Data Availability

The data that support the findings of this study are from different sources (PubMed, CINAHL, Web of Science, Embase, Cochrane library, Scopus, and The Malaria in Pregnancy Consortium Library, thesis online, Google Scholar, OpenGrey, ProQuest, ClinicalTrials.Gov, PACTR registry, and World Health Organization International Clinical trials registry platform) and are included in the list of references.

## Abbreviations

Bti: *Bacillus thuringiensis* Var israelensis
DEET: N,N-Diethyl-meta-toluamide
EIRs: Entomological inoculation rates
HBRs: Human biting rates
IRS: Indoor residual spraying
ITNs: Insecticide-treated mosquito nets
LLINs: Long-lasting insecticidal nets
LMIC: Low- and middle-income country
LSM: Larval source management
PBO: Piperonyl butoxide
RCT: Randomized controlled trial
WHO: World Health Organization
WSEBs: Window screens and eave baffles
